# Implementing a velocity-based approach to resistance training: the reproducibility and sensitivity of different velocity monitoring technologies

**DOI:** 10.1038/s41598-023-34416-0

**Published:** 2023-05-02

**Authors:** Ivan Jukic, Andrew King, Colby A. Sousa, Katarina Prnjak, Michael R. McGuigan

**Affiliations:** 1 Sport Performance Research Institute New Zealand (SPRINZ), Auckland University of Technology, Auckland, New Zealand; 2 School of Engineering, Computer and Mathematical Sciences, Auckland University of Technology, Auckland, New Zealand; 3 School of Medicine, Western Sydney University, Sydney, Australia

**Keywords:** Physiology, Health occupations

## Abstract

This study examined the reproducibility of GymAware, PUSH2 and Vmaxpro velocity monitoring devices during resistance training (RT). The sensitivity of these devices to detect the smallest changes in velocity that correspond to true changes in RT performance was also investigated. Fifty-one resistance-trained men and women performed an incremental loading (1RM) test, and two repetitions to failure tests with different loads, 72 h apart. During all repetitions, mean velocity (MV) and peak velocity (PV) were simultaneously recorded by two devices of each brand. Overall, GymAware was the most reliable and sensitive device for detecting the smallest changes in RT performance, regardless of the velocity metric used. Vmaxpro can be considered as an equivalent, cheaper alternative to GymAware for RT monitoring and prescription, but only if the MV metric is used. Caution should be exercised when using PUSH2 in practice due to their comparatively higher, unacceptable measurement error and generally low sensitivity to detect changes in RT performance. Collectively, these findings support the use of MV and PV from GymAware and MV from Vmaxpro devices for RT monitoring and prescription due to their low magnitudes of error; thus, allowing for the detection of meaningful changes in neuromuscular status and functional performance during RT.

## Introduction

Resistance training (RT) is the principal mode of training for inducing muscle strength, hypertrophy, and power adaptations. Additionally, RT has an important role in injury prevention and rehabilitation, as well as general well-being due to its beneficial effects on quality of life^1–3^. Consequently, RT is a part of many physical preparation programs of athletes and is a popular choice of exercise among recreational trainees. Several variables should be considered when designing an efficacious RT program, with RT intensity and volume receiving the greatest attention from the scientific community as they often determine the magnitude of resultant training adaptations^4,5^. RT intensity is traditionally prescribed as a percentage of an actual or estimated one-repetition maximum (1RM) assessment. On the other hand, when the number of sets for a training session is fixed, volume is prescribed by the number of repetitions performed per set (i.e., set volume). Set volume prescription is often based on the theoretical relationship between the maximum number of repetitions an individual can complete with a given percentage of 1RM. Thus, both RT intensity and volume prescription rely upon the accuracy of 1RM assessments. While 1RM tests were shown to be valid and reliable, they are physically and psychologically demanding, time-consuming, and can compromise the safety of individuals performing the test^6^. Additionally, 1RM-based RT intensity and volume prescription might be insensitive to potential day-to-day variation in performance caused by normal biological and psychological variability (e.g., sleep, nutrition, and life stress)^7,8^. This can lead to erroneous training prescriptions and sub-optimal training adaptations in the long term. To solve these fundamental issues with traditional RT intensity and volume prescription, sport scientists have proposed the use of movement velocity as a more accurate monitoring and prescription tool for RT programs^9^.

A strong, inverse relationship exists between barbell velocity and the percentage of one-repetition maximum (%1RM) in many RT exercises. Indeed, this relationship is reliable in both the Smith machine and free-weight exercises^9,10^. Thus, this relationship is useful for prescribing daily RT intensity by adjusting the absolute load to match the velocity associated with the intended %1RM depending on the trainee’s preparedness. On the other hand, Miras-Moreno et al.^11^ recently reported that movement velocity during RT can predict the maximum number of repetitions that can be completed in a set, allowing for RT set-volume prescription. Furthermore, when the exercise is performed with maximal concentric effort and fatigue ensues, velocity will decline^10^. In this regard, research has shown that monitoring velocity loss (VL) incurred in a set is an objective, practical and non-invasive indicator of the acute metabolic stress, hormonal response and mechanical fatigue induced by RT^12^. Although this velocity-based approach to RT monitoring and prescription is now widely used among different populations, several factors such as the cost of the equipment and its accuracy must be considered.

Regardless of the population in question, it is evident that the efficacy of the velocity-based approach to RT monitoring and prescription depends upon the reliability of devices used to record barbell velocity. This requirement also represents one of the main drawbacks of the velocity-based approach to RT monitoring and prescription since very small changes in velocity can represent decisive changes in neuromuscular status and functional performance^12,13^. In this regard, 3-dimensional motion capture systems are considered gold-standard for movement velocity monitoring. However, the considerable cost, space requirements, and time for data processing make motion capture systems highly impractical for most applied settings. Thanks to technological advancements, many different field-based devices such as linear position transducers (LPTs), linear velocity transducers, and inertial measurement units (IMU) have recently emerged to combat logistical constraints associated with velocity monitoring devices. One such popular device available on the market is GymAware (GymAware Power Tool; Kinetic Performance Technologies, Canberra, Australia) LPT which is also well established in the scientific literature as an accurate velocity monitoring tool during RT^14^. Although reliable and valid, several drawbacks may limit GymAware’s widespread use. Firstly, nonprofessional athletes and coaches as well as recreational trainees who have a limited budget could find it cost prohibitive. Secondly, the cable of this LPT must be attached to the barbell to record barbell velocity which may limit the number of lifting exercises it can effectively quantify. This has prompted a need for cheaper and wireless devices that could accurately record movement velocity during RT.

More recently, two wearable, wireless, IMU-based velocity monitoring devices PUSH2 (PUSH Inc., Toronto, ON, Canada) and Vmaxpro (alias EnodePro; Blaumann & Meyer—Sports Technology UG, Magdeburg, Germany) have grown in popularity among athletes and recreational trainees due to their versatility and relatively affordable price. Several studies^15–17^ have examined the test–retest reliability of PUSH2 and Vmaxpro devices with free-weight exercises. However, it is important to highlight that the test–retest reliability assessment inevitably contains errors due to biological variation. Similarly, by quantifying the degree of agreement among individuals under a single testing condition and using only one device, the true technological error of a given device cannot be discerned from the biological variation. This is pertinent during RT, where fluctuations in strength and readiness to train can cause substantial alterations in velocity outputs despite the same relative load being used. Furthermore, Bland–Altman analyses (and plots) are often used to make conclusions about the reliability of a given device. While Bland–Altman analysis is extremely useful for such purposes, it requires the interpretation of the magnitude of errors according to some practical criteria. For instance, in the context of velocity monitoring devices during RT, it would be useful to examine the sensitivity of devices to detect minimal changes in velocity that correspond to true changes in performance. However, only a few studies have based their findings on these criteria^18–20^, none of which examined the reproducibility and sensitivity of Vmaxpro and *body mode* PUSH2 devices. Therefore, research assessing the reliability of devices that account for biological variation while determining the sensitivity of devices based on practically relevant criteria is required.

**Figure 1:**
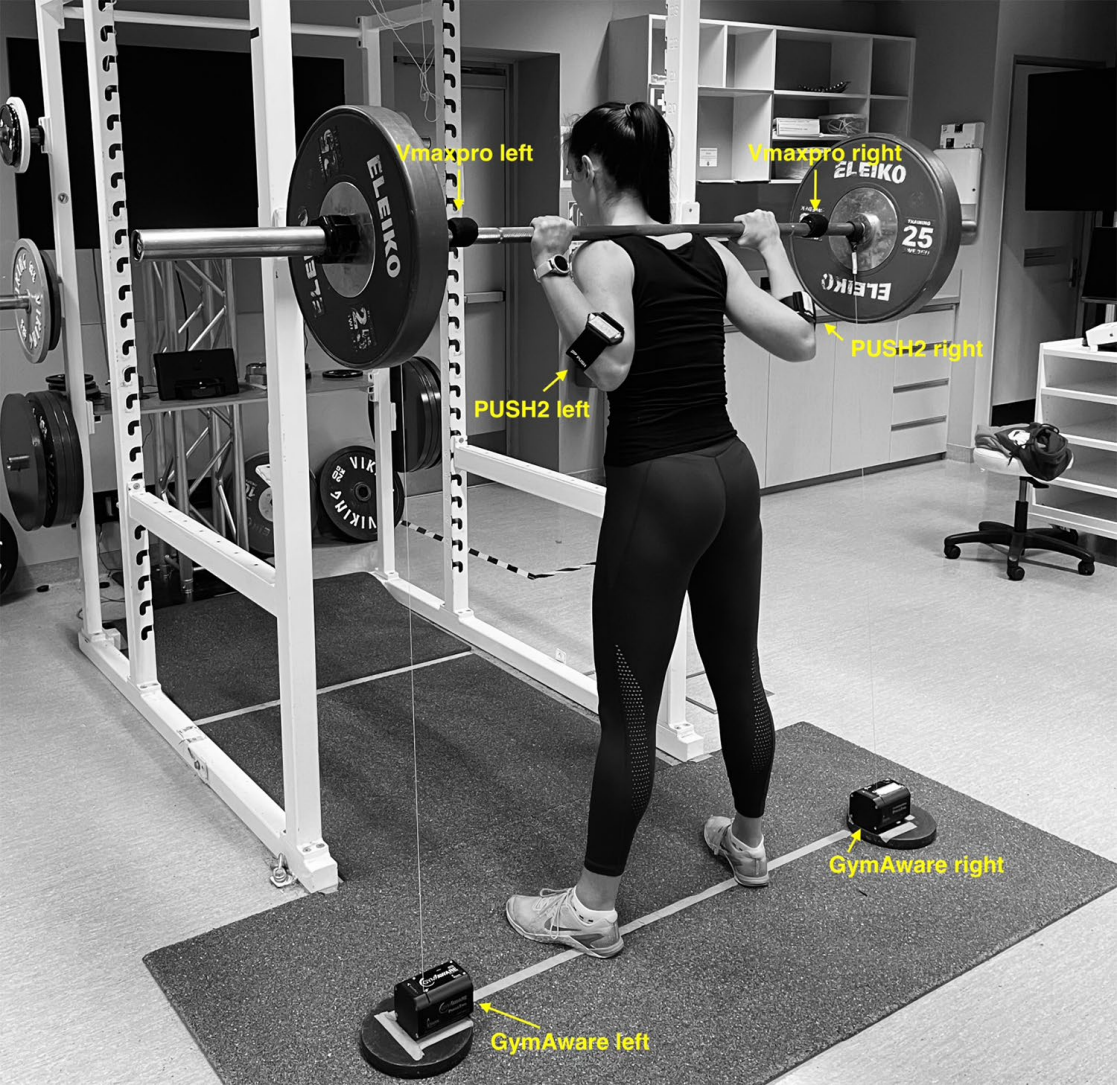
Velocity monitoring devices setup. The setup involved one GymAware and Vmaxpro device on each side of the barbell and one PUSH2 device on each forearm of the participant.

To address these shortcomings in the literature, this study aimed to comprehensively examine (1) the reproducibility of GymAware, PUSH2, and Vmaxpro devices by monitoring barbell velocity with two devices of each brand simultaneously during the free-weight back squat exercise; and (2) the sensitivity of these devices to detect minimal changes in velocity that correspond to a real change in RT performance. Such evidence is important to guide velocity-based RT monitoring and prescription and ultimately determine the most efficient and cost-effective devices for velocity monitoring during free-weight RT (Fig. 1).

## Results

**Figure 2:**
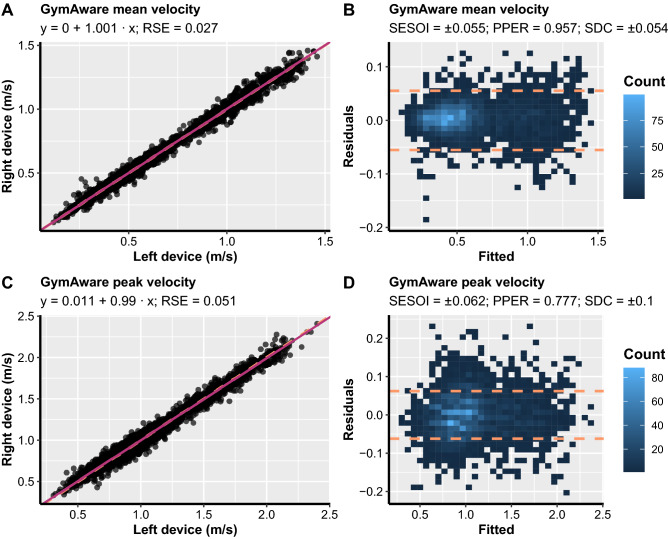
Scatterplots between left and right units (**A** and **C**) and ordinary least product (OLP) model residuals plots (**B** and **D**) for GymAware within-units comparisons. Left-hand panels show the identity line (orange dashed line) and OLP model prediction (magenta solid line). Ordinary least product model intercept (fixed bias), slope (proportional bias), and residual standard error (RSE) are listed in each panel. The residuals plot depicts a scatterplot between model fit on the x-axis and model residuals on the y-axis. The smallest effect size of interest (SESOI) area is surrounded by orange dashed lines. The proportion of practically equivalent residuals (PPER) represents the proportions of residuals that are within SESOI limits. Note: SDC, smallest detectable change.

**Figure 3:**
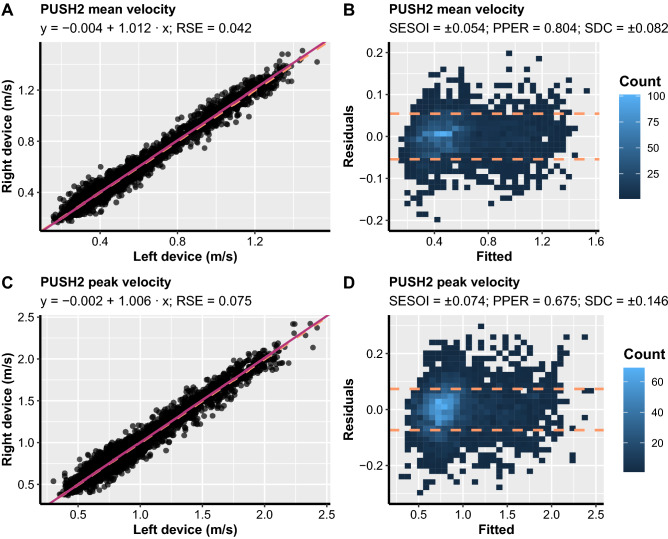
Scatterplots between left and right units (**A** and **C**) and ordinary least product (OLP) model residuals plots (**B** and **D**) for PUSH2 within-units comparisons. Left-hand panels show the identity line (orange dashed line) and OLP model prediction (magenta solid line). Ordinary least product model intercept (fixed bias), slope (proportional bias), and residual standard error (RSE) are listed in each panel. The residuals plot depicts a scatterplot between model fit on the x-axis and model residuals on the y-axis. The smallest effect size of interest (SESOI) area is surrounded by orange dashed lines. The proportion of practically equivalent residuals (PPER) represents the proportions of residuals that are within SESOI limits. Note: SDC, smallest detectable change.

**Figure 4:**
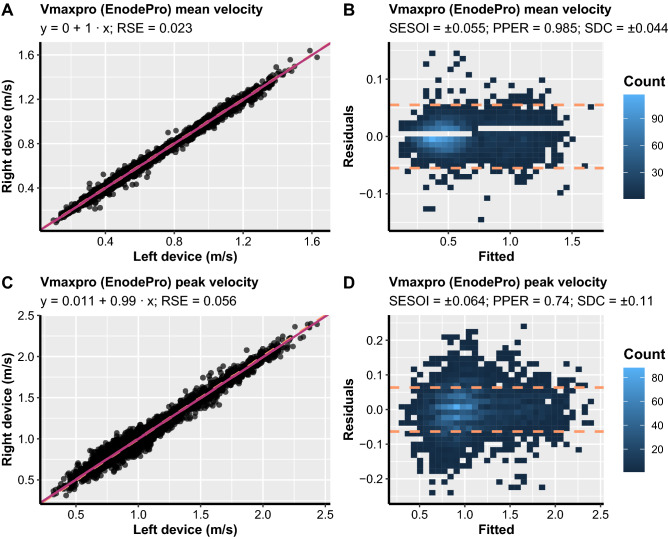
Scatterplots between left and right units (**A** and **C**) and ordinary least product (OLP) model residuals plots (**B** and **D**) for Vmaxpro (alias, EnodePro) within-units comparisons. Left-hand panels show the identity line (orange dashed line) and OLP model prediction (magenta solid line). Ordinary least product model intercept (fixed bias), slope (proportional bias), and residual standard error (RSE) are listed in each panel. The residuals plot depicts a scatterplot between model fit on the x-axis and model residuals on the y-axis. The smallest effect size of interest (SESOI) area is surrounded by orange dashed lines. The proportion of practically equivalent residuals (PPER) represents the proportions of residuals that are within SESOI limits. Note: SDC, smallest detectable change.

The scatterplots between left and right device (left-hand panels) and OLP model residuals plots (right-hand panels) for all six within-unit comparisons together with fixed bias, proportional bias, RSE, and SDC are illustrated in Figs. 2, 3, and 4. The within-unit reliability estimators and their accompanying 95% BCa confidence intervals can be found in Supplementary file III.

**Figure 5:**
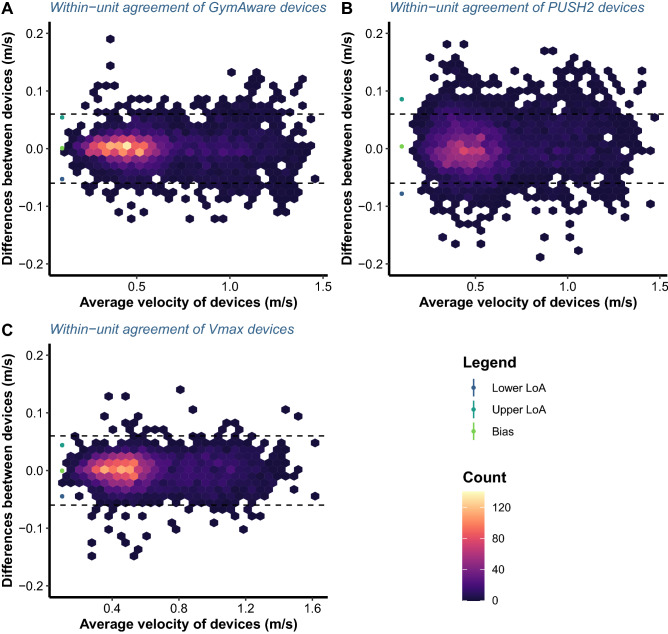
Bland–Altman plots illustrating the agreement between the repetitions mean velocity for two GymAware (**A**), PUSH2 (**B**) and Vmaxpro (alias, EnodePro) devices (**C**). Dashed lines represent an equivalent margin of ± 0.06 m/s defined as the smallest detectable change in load-velocity profiles during the free-weight back squat exercise. LoA represents limits of agreement.

For the GymAware devices, statistically significant fixed and proportional bias were observed for PV (intercept = 0.011 m/s, 95% CI [0.006, 0.017]; slope = 0.99, 95% CI [0.985, 0.995]; Figs. 2, 5), but not MV (intercept = 0 m/s, 95% CI [− 0.002, 0.002]; slope = 1.001, 95% CI [0.996, 1.005]; Figs. 2, 5). However, since the confidence intervals were within the SESOI limits (SESOI =  ± 0.062 m/s) for the PV intercept and considering that the confidence intervals were extremely narrow and very close to the value of 0 and 1 for intercept and slope, respectively, this difference could be considered practically irrelevant. Moreover, SDC for MV was within the SESOI limits for GymAware devices (SDC = 0.054 m/s). This was reflected by the SDC%1RM which was lower than the a priori selected load SESOI of 5% 1RM (SDC%1RM = 4.418%) (Figs. 2, 5). In addition, SDC for PV was outside SESOI limits (SDC = 0.100 m/s) which resulted in SDC%1RM also being above SESOI of 5% (SDC%1RM = 6.515%). Overall, MV showed perfect within-device agreement while PV showed almost excellent within-device agreement for GymAware devices during the free-weight back squat exercise.

For the PUSH2 devices, statistically significant fixed and proportional bias were observed for MV (intercept = − 0.004 m/s, 95% CI [− 0.007, 0]; slope = 1.012, 95% CI [1.006, 1.018]; Fig. 3) but not PV (intercept = − 0.002 m/s, 95% CI [− 0.009, 0.005]; slope = 1.006, 95% CI [0.999, 1.014]; Fig. 3). However, since the confidence intervals for the MV intercept were within the SESOI limits (SESOI = ± 0.054 m/s) and considering that the confidence intervals were extremely narrow and very close to the value of 0 and 1 for intercept and slope, respectively, this difference could be considered practically irrelevant. SDC for both MV (0.082 m/s) and PV (0.146 m/s) were outside the SESOI limits for the PUSH2 devices (SESOI = ± 0.054 m/s for MV; and SESOI = ± 0.074 m/s for PV) which resulted in SDC%1RM also being above SESOI of 5% (SDC%1RM ≥ 6.687%). Overall, while both MV and PV of PUSH2 devices do not seem to be affected by fixed and proportional bias, they do, however, possess higher SDC than the a priori selected load SESOI of 5%, especially PV.

For the Vmaxpro devices, statistically significant fixed and proportional bias were observed for PV (intercept = 0.011 m/s, 95% CI [0.005, 0.017]; slope = 0.99, 95% CI [0.986, 0.995]; Fig. 4), but not MV (intercept = 0 m/s, 95% CI [− 0.002, 0.001]; slope = 1, 95% CI [0.996, 1.003]; Fig. 4). However, since the confidence intervals were within the SESOI limits (SESOI =  ± 0.062 m/s) for the PV intercept and considering that the confidence intervals were extremely narrow and very close to the value of 0 and 1 for intercept and slope, respectively, this difference could be considered practically irrelevant. SDC for MV was also within the SESOI limits for the Vmaxpro devices (SDC = 0.044 m/s). This was reflected by the SDC%1RM which was lower than the a priori selected load SESOI of 5% 1RM (SDC%1RM = 3.607%). In addition, SDC for PV was outside SESOI limits (SDC = 0.11 m/s) which resulted in SDC%1RM also being above SESOI of 5% (SDC%1RM = 6.907%). Overall, both MV and PV of Vmaxpro devices do not seem to be affected by fixed and proportional bias. However, only MV showed perfect within-device agreement since PV displayed higher SDC than the a priori selected load SESOI of 5%.

**Figure 6:**
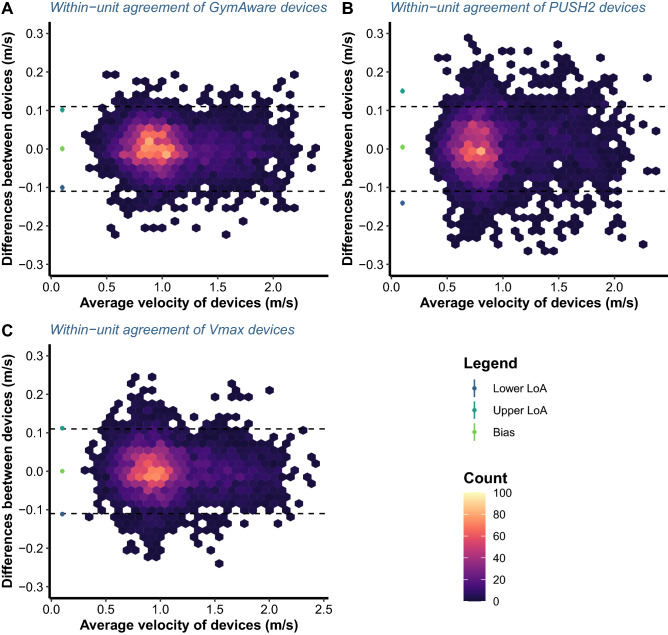
Bland–Altman plots illustrating the within-unit agreement between the repetitions peak velocity for two GymAware (**A**), PUSH2 (**B**) and Vmaxpro (alias, EnodePro) devices (**C**). Dashed lines represent an equivalent margin of ± 0.11 m/s defined as the smallest detectable change in load-velocity profiles during the free-weight back squat exercise. LoA represents limits of agreement.

The relative reliability (i.e., ICCs) of all devices, as well as their concordance (i.e., CCC), was good to excellent, whereas absolute reliability (i.e., CVs) was lower for PUSH2 compared to GymAware and Vmaxpro, and higher than the acceptable threshold of 5% (Table 1). The null hypothesis for the equivalence of MV of repetitions recorded by the left and right devices was rejected for GymAware and Vmaxpro, but not PUSH2 devices since the 1–2α confidence intervals of LoA were completely within the ± equivalent margin of 0.06 m/s (Fig. 5; Table 1). For PV, the null hypothesis for the equivalence was only rejected for GymAware devices due to 1–2α confidence intervals of LoA being completely within the ± 0.11 m/s margin of equivalence, as opposed to the other two devices (Fig. 6; Table 1).

**Table 1:** Estimates of complementary statistical parameters and parameters estimated by Bland–Altman analysis with 95% confidence intervals.

Device	Variable	CV	ICC	CCC	Bias	Upper LoA	Lower LoA
GymAware	Mean velocity	3.358	0.995	0.995	0.001	0.054	− 0.053
		[3.286, 3.433]	[0.995, 0.995]	[0.995, 0.995]	[− 0.002, 0.004]	[0.051, 0.576]	[− 0.056, 0.050]
	Peak velocity	3.405	0.989	0.989	0.001	0.102	− 0.1
		[3.333, 3.482]	[0.989, 0.990]	[0.989, 0.990]	[− 0.006, 0.007]	[0.095, 0.109]	[− 0.108, − 0.094]
PUSH2	Mean velocity	5.141	0.987	0.987	0.004	0.086	− 0.078
		[5.027, 5.261]	[0.987, 0.988]	[0.987, 0.988]	[0.001, 0.007]	[0.082, 0.089]	[− 0.081, − 0.074]
	Peak velocity	5.64	0.979	0.979	0.005	0.15	− 0.141
		[5.522, 5.780]	[0.978, 0.980]	[0.978, 0.980]	[− 0.002, 0.011]	[0.144, 0.157]	[− 0.147, − 0.134]
Vmaxpro (EnodePro)	Mean velocity	2.722	0.997	0.997	− 0.001	0.044	− 0.045
		[2.663, 2.784]	[0.996, 0.997]	[ 0.996, 0.997]	[− 0.002, 0.002]	[0.042, 0.046]	[− 0.471, − 0.042]
	Peak velocity	3.763	0.987	0.988	0.001	0.112	− 0.111
		[3.681, 3.848]	[0.987, 0.988]	[0.987, 0.988]	[− 0.005, 0.006]	[0.106, 0.118]	[− 0.117, − 0.106]

*[number, number]* confidence interval, *CV* coefficient of variation, *ICC* intraclass correlation coefficient, *CCC* Lin’s concordance correlation coefficient, *LoA* level of agreement.

## Discussion

To our knowledge, this is the first study that examined the reproducibility of GymAware, PUSH2 and Vmaxpro velocity monitoring devices during the free-weight back squat exercise while also examining their sensitivity for detecting true changes in RT performance. The main findings of this study were (1) fixed and proportional bias was not observed for any of the devices and velocity metrics examined in this study; (2) only MV from GymAware and Vmaxpro, but not PUSH2 devices were sensitive enough to detect true changes in RT performance according to the a priori established criteria; (3) GymAware (MV and PV) and Vmaxpro (MV), but not PUSH2 (neither MV nor PV) devices demonstrated high levels of within-unit agreement (i.e., equivalence) concerning SDC for load-velocity profiles in the free-weight back squat exercise previously reported in the literature. Collectively, these findings support the use of MV and PV from GymAware LPT and MV from Vmaxpro devices for RT monitoring and prescription due to their low magnitudes of error; thus, allowing for sensible detection of meaningful changes in neuromuscular status and functional performance during RT.

Most of the available studies examining the reliability of velocity monitoring devices including PUSH2 and Vmaxpro have quantified inter-individual or intra-individual variability in movement velocity during the same testing condition (e.g., velocity against the same relative load for all individuals) or during the “same” testing condition separately by a specific amount of time (e.g., 48–72 h; test–retest reliability), respectively. However, this study design fails to separate biological (i.e., human) and technological variation and, as such, does not reveal any information about the true source of measurement error^14^. When the calibration device is not available, an effective approach to examine devices’ reproducibility (i.e., their true technological error) is to simultaneously use two or more devices of the same brand under the same testing conditions^18–20^. This approach was taken in the current study and revealed that GymAware devices possess almost perfect reliability for MV as evidenced by low RSE, high PPER and sensitivity to detect meaningful changes in performance (i.e., SDC%1RM), and the absence of fixed and proportional bias. In comparison, PV from GymAware devices demonstrated small, but practically non-significant fixed and proportional bias, and slightly lower PPER and SDC%1RM. These findings are in partial agreement with the only previous study^18^ examining the reproducibility of GymAware while simultaneously using two devices under the same testing conditions. More specifically, Jovanovic and Jukic^18^ found PV from GymAware to be a more sensitive metric compared to MV in detecting changes in performance during the hex-bar deadlift exercise, although both velocity metrics were found to be highly sensitive and reliable. It is important to highlight that although PV from GymAware was less sensitive than MV in the present study, it was still sensitive enough to detect meaningful changes in RT performance. Namely, when investigating the equivalence of PV recorded by two GymAware devices, upper and lower LoA were within the lowest SDC for the individualised load-velocity profiles reported by Banyard et al.^10^ during the free-weight back squat exercise. Therefore, it can be concluded that both MV and PV from GymAware are reliable and sensitive to detecting meaningful changes in neuromuscular status and functional performance during the free-weight back squat exercise.

In the present study, fixed and proportional bias was not an issue for *body mode* PUSH2 devices. However, somewhat higher RSE, PPER and SDC%1RM compared to GymAware—especially for MV—and a priori established criteria, suggest that caution should be exercised when using PUSH2 devices in practical settings. In the previous study^18^, where the reproducibility of bar mode PUSH2 devices was examined, the authors reported worse RSE, PPER, and SDC%1RM than those observed in the present study for the PUSH2 device. Therefore, although the reproducibility of *body mode* PUSH2 devices examined in the present study was unacceptable, reproducibility was greater than for previously reported *bar mode* PUSH2 devices. It is important to note that several software updates may have played a bigger role in the improved reproducibility of the PUSH2 devices observed in the present study compared to the differences between the *body* and *bar mode* of PUSH2 devices. Furthermore, we also observed a lack of agreement for both MV and PV between two PUSH2 devices concerning the SDC of the individualised load-velocity profiles in the free-weight back squat exercise. Therefore, while substantial fixed and proportional bias was not present for PUSH2 devices, they do seem to possess a considerable amount of random error which negatively affects their sensitivity in detecting practically relevant changes in performance regardless of the velocity metric used. Nevertheless, it should be noted that MV from PUSH2 had a reasonably high PPER and low SDC%1RM, suggesting that practitioners who are only interested in detecting changes in performance greater than ~ 7% 1RM could still benefit from using PUSH2 devices. Though, caution should again be exercised when using these devices for monitoring neuromuscular status or terminating training sets based on velocity loss.

For Vmaxpro devices, neither fixed nor proportional bias was observed in the present study. Importantly, very low RSE and SDC%1RM, and high PPER were observed for MV. Interestingly, these values for RSE, SDC%1RM, and PPER were superior in comparison to GymAware and PUSH2 devices. In addition, MV between the two Vmaxpro devices was highly equivalent concerning SDC in load-velocity profiles suggesting their sensitivity in detecting very small changes in performance. However, RSE and SDC%1RM for PV were twice as high compared to MV of this device, but still slightly superior to those of PUSH2, but not GymAware devices. This finding was further reflected by the equivalence analysis for PV of Vmaxpro where upper and lower LoA always sat just outside (~ 0.007 m/s) the practically equivalent margin of ± 0.11 m/s which corresponds to a real change in daily readiness to train during the free-weight back squat exercise when assessed by PV^10^. Therefore, MV from Vmaxpro devices can be used for RT monitoring, prescription, and performance evaluation, whereas caution should be exercised when using PV from Vmaxpro as it does not seem to be sensitive enough to detect the smallest changes in performance that could be relevant in practical settings.

Traditional interpretations of correlations and linear relationship coefficients (i.e., values > 0.90 as very high) previously failed to confirm devices’ reliability^19,20^. Similarly, in the present study, MV and PV between pairs of the same device all showed high *r* values (i.e., ~ 0.98). However, this just means that the faster the movement on the first device, the faster the movement on the second device, giving no information about the magnitude of errors in absolute values. Since velocity monitoring devices, among other purposes, are used to establish the load-velocity profiles for the individuals it is crucial to evaluate their magnitudes of error in both absolute (i.e., m/s) and relative, practical terms (i.e., %1RM), as done in the present study. Furthermore, stricter criteria than previously used should be adopted to assess technological variability, as recently recommended by Courel-Ibáñez et al.^19^ For instance, if one considers CV values of <10% and ICC values> 0.90 to represent good reliability of a given device, then one also must accept the remaining 10% error in the measurement. While these criteria could be seen as more than rigorous in social sciences, it is not enough for the assessment of technological devices. In this regard, Courel-Ibáñez et al.^19^ suggested ICC > 0.99, CV < 3.5%, RSE < 0.03 m/s for velocity monitoring devices to be considered reliable and possess acceptable sensitivity. Following these recommendations, only MV and PV from GymAware and MV from Vmaxpro can be used for RT monitoring and prescription, as previously concluded based on other statistical parameters provided in the present study. Finally, future research should also consider evaluating a wide range of statistical parameters rather than relying on traditional correlation, CV, and ICC coefficients to provide more nuanced insights into the reliability and sensitivity of velocity monitoring devices.

In the present study, the reproducibility of GymAware, *body mode* PUSH2 and Vmapro devices during the free-weight back squat exercise while evaluating and interpreting the magnitudes of errors according to pre-established, practical criteria. A relatively large number of males and females with different training backgrounds and experience levels performed ecologically valid RT sets with a range of different loads and repetition ranges. However, it should be noted that the validity of these devices was not evaluated in the current study due to the absence of a gold standard (i.e., the MOCAP system). In addition, the validity of GymAware, PUSH2 and Vmaxpro devices has been examined in previous studies using different approaches with conflicting findings reported, especially for PUSH2 and Vmaxpro devices^16,17^. Therefore, future research is required to comprehensively examine the validity of these devices. Nevertheless, if the reproducibility and sensitivity of a given device are proven sound, the practitioners can be confident that the measurements are sensitive enough to detect practically meaningful changes in barbell velocity. While we aimed to isolate technological error by simultaneously using two devices of the same brand under the same testing conditions, it must be noted that a biological source of error could still be introduced in this setting if any side-to-side differences in the displacement of the ends of the barbell occur during free-weight RT exercises. To our knowledge, no study has quantified the effects of the barbell dip on the magnitude of this error. Therefore, it cannot be determined whether this affected the reproducibility and sensitivity analyses of the devices examined in the present study. Nevertheless, to circumvent the potential limitation of simultaneously using two devices of the same brand under the same testing conditions to examine their reproducibility and sensitivity, we recommend future researchers change the devices’ position to the opposite end of the barbell after each set. Finally, it should be noted that manufacturers of velocity monitoring devices are frequently releasing new software updates for devices and increasing the accuracy and stability of the algorithms used. This further suggests that the reproducibility of devices should be examined regularly to ensure that the measurements obtained are reliable and sensitive enough for the intended purpose.

The findings of the present study suggest that GymAware is highly reproducible and sensitive in detecting the smallest changes in RT performance, regardless of the velocity metric used. Vmaxpro can be considered as an equivalent, cheaper alternative to GymAware, but only if the MV metric is used for RT monitoring and prescription since PV from Vmaxpro was found to be a slightly less sensitive metric compared to PV from GymAware. Caution should be exercised when using PUSH2 devices in practice due to their comparatively higher, unacceptable measurement error and generally low sensitivity to detecting meaningful changes in neuromuscular status and functional performance during a free-weight back squat exercise.

## Methods

### Study design

This study was part of a larger investigation on the reliability of several different velocity-based monitoring and prescription methods for resistance training. Participants made five visits to the laboratory, with each visit separated by 48–72 h. The first session familiarised participants with the free-weight back squat movement, the equipment used during the experimental sessions, the instruction to move the barbell up as fast as they can during the concentric phase, and visual feedback indicating the velocity of the barbell on a screen (Fig. 1). At the subsequent two sessions, participants completed a 1-repetition maximum test in the free-weight back squat. In the final two sessions and using the 1-RM load obtained from the second 1-RM session, participants completed a repetition to failure (RTF) test in which they performed sets to failure in the back squat exercise with 90, 80, and 70% of their pre-determined 1-RM. Mean velocity (MV) and peak velocity (PV) were recorded by two Gymaware, PUSH2, and Vmaxpro devices during all testing sessions.

### Participants

Fifty-one resistance-trained participants (15 females and 36 males; 18 to 40 years of age; back squat 1RM/body mass = 1.24 ± 0.32 and 1.79 ± 0.35 for females and males, respectively) completed this study. To be eligible for inclusion, participants (a) abstained from additional lower-body training during their participation in the study; (b) were not taking medication known to alter metabolic or cardiovascular function; (c) were free of musculoskeletal injury; (d) were not reportedly using, or had a history of using anabolic steroids; and (e), had at least six months of resistance training experience in the back squat exercise, including at least two sessions per week and one performing the back squat, and no longer than two weeks without training in this period. Participants provided informed written consent before commencing the study. The study protocol was approved by the Auckland University of Technology Ethics Committee (approval number: 20/55), and The Code of Ethics of the World Medical Association. All experiments were performed in accordance with relevant guidelines and regulations.

### Familiarisation session

Participants’ body mass and height were recorded using an electronic column scale and a wall-mounted stadiometer (Seca Ltd., Hamburg, Germany). Thereafter, participants completed a standardised warm-up consisting of cycling at 100 rpm for 5 min; dynamic stretching for 2 min; 10 bodyweight lunges and squats; and 10 barbell squats. After the warm-up, participants were familiarised with the instruction to lift the barbell up as fast as they can during the concentric phase of the squat, and visual velocity feedback (using the right GymAware) displayed on a TV screen in front of them (~ 3 m) indicating the velocity of the barbell, and the instruction to have at least a momentary pause (no longer than 2 s) between the repetitions. Participants then completed 3 repetitions at 20, 40, and 60% of their estimated 1RM, and 10 repetitions at 60% of their estimated 1RM. Participants practised lifting the barbell up as fast as they can, avoided pausing more than 2 s between repetitions, and received visual feedback indicating the velocity of the barbell. At the end of each session, all participants understood and felt comfortable with these conditions, and had done at least two sets with very consistent velocities of the repetitions (± 0.02 m/s). To inform warm-up loads for the upcoming 1-RM sessions, participants were also asked to provide the log of their most recent (and heaviest) back squat session and to conservatively estimate their 1RM.

### One repetition maximum sessions: Days 2–3

Participants completed the same standardised warm-up as in the familiarisation session. A 20-kg barbell (Rogue, Columbus, Ohio, USA) and calibrated weight plates (Eleiko; Halmstad, Sweden, EU) were used. The 1RM protocol consisted of 3 repetitions at 20%, 3 repetitions at 40%, 3 repetitions at 60%, 1 repetition at 80%, and 1 repetition at 90% of an estimated 1RM, followed by 1RM attempts. After each successful attempt, the load was increased in consultation with the participant, using increments of 1 to 12.5 kg until no further weight could be lifted or until the movement technique was compromised. A maximum of five 1RM attempts were allowed for each participant. Three and four minutes of passive rest were provided between each submaximal set and 1RM attempt, respectively. Each participant adopted a shoulder-width stance and used a self-regulated eccentric velocity. Immediately upon reaching the bottom of their squat, participants were instructed to perform the concentric (upward) portion of each repetition as fast as possible. Verbal encouragement and visual feedback (indicating the velocity of the barbell) were provided throughout all trials. Participants were required to reach a depth of the squat at which the top of the thighs was at least parallel to the floor, as determined by the investigators and a camera positioned perpendicularly to the participant, for the repetition to be considered successful. During all repetitions, the feet were required to maintain contact with the floor (i.e., no jumping or lifting of the heels) and a slight pause was required after each repetition with full hip and knee extension. The rest between 1RM sessions was 48 h.

### Repetitions to failure sessions: Days 4–5

Participants completed the same standardised warm-up as in the familiarisation and 1RM sessions. Thereafter, they completed four sets of 10, 5, 3, and 1 repetition against 30%, 50%, 70%, and 90% of the 90% of their established 1RM (i.e., the heaviest load to be lifted that day), respectively. Participants were provided 3 min of rest between warm-up sets, and 4 min between the last warm-up set and the first set to failure. Thereafter, participants performed three sets with 90, 80 and 70% 1RM respectively, to muscular failure with 10 min of inter-set rest. Since the excessive fatigue caused by performing a high number of repetitions during RTF with lower loads (i.e., 70% 1RM) could have compromised the number of repetitions performed during subsequent RTF sets with greater loads (i.e., 80% and 90% of 1RM), the order of loads was not randomised. Instead, participants always performed RTF with the highest load (i.e., 90% 1RM) first while the last RTF set was always performed with the lowest load (i.e., 70% 1RM). Regarding the exercise execution (including lifting instructions, encouragement, and visual feedback), the same conditions were applied as during 1RM sessions. The rest between RTF sessions was 72 h.

### Data acquisition

A comprehensive description of data acquisition is provided in Supplementary File I. Briefly, two GymAware, PUSH2, and Vmaxpro (alias, EnodePro) devices were used at the same time to measure MV and PV during all repetitions throughout the sessions. One GymAware and one Vmaxpro device were attached to the left and the right side of the barbel as shown in Fig. 1. In contrast, one PUSH2 device was placed on each forearm (one device on each forearm) with the hand supinated, on top of the ulna, 1–2 cm distal to the elbow, and with the main button located proximally according to the manufacturer’s instructions (Fig. 1).

Data obtained from GymAware and Vmaxpro were transmitted via Bluetooth to a tablet (iPad, Apple Inc., California, USA) using the GymAware v2.8.0 app and Vmaxpro app v4.2.0, respectively. Prior to each measurement, the Vmaxpro devices were calibrated according to the manufacturer’s instructions. Vmaxpro devices were placed on both sides of the barbell between the hands and the loaded barbell sleeves (using a Velcro strap) next to Gymaware’s cables. Data obtained from PUSH2 were transmitted to the PUSH v7.6.0 app via Bluetooth connection with two smartphones (iPhone, Apple Inc., California, USA). The same two researchers recorded all MV and PV data in Microsoft Excel (Microsoft Corporation, Redmond, Washington, USA) during each session. Each device was labelled with the words “left” and “right” and was consistently used on their respective sides of the barbell or forearm. In addition, each app, for every respective device was run with iOS 14.0.1. The barbell was marked so that the positioning of the devices could be kept identical throughout all trials for all participants.

### Statistical analysis

All data were normally distributed as determined by the graphical inspection and the indicator value range for skewness and kurtosis. Descriptive data are presented as Means and SDs unless otherwise stated. To examine within-unit reliability (right vs left device for each measuring device separately) for MV and PV, ordinary least products (OLP) regression was used. Estimated parameters of the OLP regression (intercept, slope, and residual standard error [RSE]) were used for further analysis, where the intercept and slope were considered to represent fixed and proportional bias, respectively. If the 95% confidence interval for the intercept did not include 0, then fixed bias was present. If the 95% confidence interval for the slope did not include 1, then proportional bias was present. To judge the practical significance of the within-device reliability, the smallest effect size of interest (SESOI) was used^21^. In this study, SESOI was defined as the minimal change in the load that corresponds to ± 5% 1RM. To estimate velocity SESOI (for both MV and PV), pooled load-velocity profile was used to estimate the average change in velocity that corresponds to a change in the load of ± 5% 1RM.

To judge the magnitude of error, the proportion of practically equivalent residuals (PPER), smallest detectable change in velocity (SDC) and %1RM (SDC%1RM) were evaluated^18^. Statistical inference for all within-unit agreement parameters was provided using the stratified 12,000 resamples bootstrap and 95% bias-corrected and accelerated (BCa) confidence intervals. A set of complimentary statistical parameters including Pearson correlation coefficient (r), intra-class correlation coefficient (ICC), coefficient of variation (CV) and Lin’s concordance correlation coefficient (CCC) with 95% confidence intervals (CI) were also evaluated.

To examine whether the devices of the present study are sensitive enough to detect changes in load-velocity profiles (LVP) previously reported for the free-weight back squat exercise^10^, a modified true value varies method of the Bland–Altman analysis for multiple observations per participant was used^22^. The bias and associated 95% limits of agreement (LoA) were evaluated and interpreted in the context of an a priori specified equivalent margin of ± 0.06 m/s and 0.11 m/s for MV and PV, respectively. These criteria were selected since they represent the SDC in velocity for individual LVPs in the free-weight back squat exercise^10^. All assumptions of the Bland–Altman analysis were satisfied. All analyses were done with a custom-written script in R statistical language, that is available together with the dataset at the Open Science Framework repository (https://osf.io/cbsar/).

Three participants withdrew from the study due to injuries during their work or recreational sporting activity not related to the study whereas two participants dropped out of the study due to personal reasons after completing one or three experimental sessions. However, their data was not removed from the analyses. The number of participants, total repetition observations, missing observations for each measuring unit per day and testing protocol are presented in Supplementary File II. More information regarding (a) devices used and their software, (b) calculation of statistics relating to the magnitude of error, (c) statistical inference using stratified bootstrap, (d) Bland–Altman analysis, and (e) missing data points is provided in Supplementary File I.

## Supplementary Information

Supplementary Information 1.

Supplementary Information 2.

Supplementary Information 3.

Supplementary Information 4.

## Data Availability

The datasets generated during the current study are available in the Open Science Framework repository, https://osf.io/cbsar/.
