# Supplementary material for: Implementing a velocity-based approach to resistance training: the reproducibility and sensitivity of different velocity monitoring technologies
Source: Sci Rep. 2023 May 2;13:7152. doi: 10.1038/s41598-023-34416-0 (PMC10154341; doi:10.1038/s41598-023-34416-0)
Supplement: Supplementary file 1 — Supplementary Information 1. [file 41598_2023_34416_MOESM1_ESM.docx]

Jukic et al. (2022). Velocity-based approach to resistance training: the reproducibility and sensitivity of commercially available velocity monitoring technologies. *Scientific Reports*. Email corresponding author: ivan.jukic@aut.ac.nz. Sport Performance Research Institute New Zealand (SPRINZ), Auckland University of Technology, Auckland, New Zealand.

**Supplementary File I**

*Data acquisition – extended information*

In the present study, two GymAware, PUSH2, and Vmaxpro devices were used at the same time to measure mean (MV) and peak velocity (PV) during all repetitions throughout the sessions. One GymAware and one Vmaxpro device were attached to the left and the right side of the barbel as shown in Figure 1. In contrast, one PUSH2 device was placed on each forearm.

The GymAware is a commercially available linear position transducer consisting of a power tool, made up of a steel cable that is wound on a cylindrical spool coupled to the shaft of an optical encoder. The power tool units were placed on both sides of the barbell perpendicular to the position between the hands and the loaded barbell sleeves, according to the manufacturer’s instructions. The end of the cable was vertically attached to the barbell using a Velcro strap. The GymAware of the current study measures the total displacement of its cable in response to changes in the barbell position and incorporates an angle sensor that accounts for motion in the horizontal direction during predominantly vertical displacement measurements. The software later accounts for the total distance and angle, and using basic trigonometry, provides a resultant vertical displacement. Instantaneous velocity was determined as the change in barbell position with respect to time, which is also provided by GymAware’s software. Data obtained from GYM were transmitted via Bluetooth to a tablet (iPad, Apple Inc., California, USA) using the GymAware v2.8.0 app. Microsoft Excel (Microsoft Corporation, Redmond, Washington, USA) and prepared for further analysis.

The PUSH2 is a both wearable and attachable inertial sensor consisting of a three-axis accelerometer and a gyroscope that provides six degrees in its coordinate system. In the present study, two PUSH2 devices were strapped to participants’ forearms (one device on each forearm) with the hand supinated, on top of the ulna, 1–2 cm distal to the elbow, and with the main button located proximally according to manufacturer’s instructions (Figure 1). The acceleration data were smoothed using a Butterworth filter, and vertical velocity was calculated by the integration of acceleration with respect to time. Data obtained from the PUSH2 were recorded at a sampling rate of 1000 Hz and transmitted (while being down sampled to 200-230 Hz) to the PUSH v7.6.0 app via Bluetooth connection with two smartphones (iPhone, Apple Inc., California, USA).

The Vmaxpro, alias EnodePro is a commercially available wireless IMU that includes a three-axis accelerometer, and gyroscope. The data were directly obtained by the integration of the vertical acceleration with respect to time at a sampling rate of 1000 Hz through a Bluetooth (65 Hz) connection with a tabled (iPad, Apple Inc., California, USA) using the Vmaxpro app v4.2.0. Before each measurement, the Vmaxpro devices were calibrated according to the manufacturer’s instructions. More specifically, the devices were placed on all six sides on a plane horizontal ground. Based on this calibration, the devices adjust to environmental parameters and reach the highest accuracy based on the manufacturers’ claims. Thereafter, Vmaxpro devices were placed on both sides of the barbell between the hands and the loaded barbell sleeves (using a Velcro strap) next to Gymaware’s cables.

Each device was labelled with the words “left” and “right” and was consistently used on their respective sides of the barbell or forearm. In addition, each app, for every respective device was run with iOS 14.0.1. The barbell was marked so that the positioning of the devices could be kept identical throughout all trials for all participants. Finally, to avoid any data loss due to issues with online clouds or internet connection, MV and PV of all repetitions were manually recorded and organised in the Microsoft Excel spreadsheet (Microsoft Corporation, Redmond, Washington, USA) during each session. To ensure consistency and accuracy of this procedure, the same two researchers were handling this task throughout the study.

*SESOI calculation – extended information*

Pooled load-velocity profile was estimated using the right device of each measuring unit with the data collected during both 1RM testing protocols. Importantly, the load-velocity profile used the data from all sets, except the 1RM attempt due to its compromised reliability previously reported in the literature.^1^ In addition, for sets where more than 1 repetition was performed (i.e., sets against 20, 40, and 60% of 1RM), only the fastest repetition was used for the SESOI estimation. Supplementary file IV illustrates all the data from both testing protocols pooled together with the accompanying regression line for MV, which represents a pooled load-velocity profile. Velocity SESOI was calculated by multiplying the linear regression slope coefficient by ± 5. As such, velocity SESOI represents the average change in velocity when the load changes for ± 5% 1RM. Finally, SESOI calculation and subsequent analysis of all within-unit agreement estimators were performed for each day separately. However, since the overall estimators or conclusions of analyses did not change, pooled analysis was retained in the manuscript due to simplicity and space limitations.

*Calculation of statistics relating to the magnitude of error*

The proportion of practically equivalent residuals (PPER) was calculated using the estimated RSE and SESOI and represents the proportion of residual standard error (RSE) that is within the smallest effect size of interest (SESOI) limits. PPER estimator can take values from 0 to 1 with values closer to 1 indicating higher practical agreement. PPER was calculated assuming a normal distribution of the residuals. The smallest detectable change (SDC) was calculated using the following equation: RSE × 1.96 representing the smallest, true change in velocity rather than a measurement error 95% of the time. The smallest detectable %1RM change (SDC%1RM) was calculated by multiplying the estimated SDC by the slope coefficient of the pooled load-velocity profile. This estimator represents a true change in %1RM rather than a measurement error 95% of the time. The lower the SDC%1RM, the higher the sensitivity to detect changes in load (i.e., %1RM). A given measuring unit could be considered to possess excellent sensitivity to detect changes in load if its SDC%1RM is below its estimated SESOI (i.e., 5%).

*Statistical inference using stratified bootstrap*

Stratified bootstrap was performed because velocity SESOI estimation utilised data from 1RM protocols only (using all sets except the 1RM attempt due to its compromised reliability, previously reported in the literature^1^, whereas all data from both 1RM and RTF protocols were used for the within-unit agreement analysis.^3^ Stratified bootstrap ensures that an equal number of resamples are taken from two strata: strata for velocity SESOI estimation and strata involving all other observations. Missing observations were removed pairwise for each analysis within the bootstrap loop.

*Criteria for a set of complementary statistical parameters*

A set of complimentary statistical parameters including Pearson correlation coefficient (r), intra-class correlation coefficient (ICC), coefficient of variation (CV) and Lin’s concordance correlation coefficient (CCC) with 95% confidence intervals were also evaluated. For the assessment of technological equipment, the cut-off values for ICC (type 1, one-way random effects, absolute agreement model) of 0.95-0.99 are considered good for research and clinical practice.^5^ CCC represents how close the pairs of velocity measurements between devices are to the best-fit line, and how far this line is from the 45 degrees concordance line through the origin.^4^ A CCC value of 1 represents perfect, > 0.99 almost perfect, from 0.95 to 0.99 good, from 0.90 to 0.95 moderate, and < 0.90 poor concordance between measurements, respectively. Finally, CV < 5% was considered acceptable as reliable exercise performance tests often have CV lower than this threshold.^2^

*Bland-Altman analysis – extended information*

The confidence intervals for limits of agreement (LoA) were calculated according to the method of variance estimates recovery (MOVER) method, which considers the repeated measurements taken.^7^ In addition, for LoA, equivalence tests were also performed via two one-sided tests (TOST). The TOST procedure was performed with an α-value of 0.1 and a 1─2α confidence interval. The null hypothesis of TOST was that the two values were not equivalent. If the 1–2α confidence interval was completely contained within the ± equivalent margin, the null hypothesis was rejected, and the two sets of measurements (i.e., left, and right devices) were considered equivalent.^6^

*Missing data points*

In total, PUSH2 devices had 332, VmaxPRO had 205, and GYM had 2 missing observations. However, it should be noted that Bluetooth issues were experienced in the first few sessions in the study which probably caused missing repetitions from PUSH2 (207 observations) and VmaxPRO (21 observations) devices. In this regard, the total number of missing observations which were not related to a known issue (i.e., missing at random) was 125 and 184 for PUSH2 and VmaxPro devices, respectively.

1. Banyard H. G., Nosaka K., Vernon A. D., Haff G. G. The reliability of individualized load–velocity profiles. *Int J Sports Physiol Perform*. 2018;13(6):763-769.

2. Hopkins W. G. Measures of reliability in sports medicine and science. *Sports Med*. 2000;30(1):1-15.

3. Jovanovic M., Jukic I. Within-unit reliability and between-units agreement of the commercially available linear position transducer and barbell-mounted inertial sensor to measure movement velocity. *J Strength Cond Res*. 2020 [aop].

4. Lin L., Hedayat A., Sinha B., Yang M. Statistical methods in assessing agreement: Models, issues, and tools.*J Am Stat Assoc*. 2002;97(457):257-270.

5. Martins W., Nastri C. Interpreting reproducibility results for ultrasound measurements. *Ultrasound Obstet Gynecol*. 2014;43(4):479-480.

6. Schuirmann D. J. A comparison of the two one-sided tests procedure and the power approach for assessing the equivalence of average bioavailability. *J Pharmacokinet Pharmacodyn*. 1987;15(6):657-680.

7. Zou G. Confidence interval estimation for the Bland–Altman limits of agreement with multiple observations per individual. *Stat Methods Med Res*. 2013;22(6):630-642.
