# Supplementary material for: Implementing a velocity-based approach to resistance training: the reproducibility and sensitivity of different velocity monitoring technologies
Source: Sci Rep. 2023 May 2;13:7152. doi: 10.1038/s41598-023-34416-0 (PMC10154341; doi:10.1038/s41598-023-34416-0)
Supplement: Supplementary file 2 — Supplementary Information 2. [file 41598_2023_34416_MOESM2_ESM.docx]

Jukic et al. (2023). Implementing a velocity-based approach to resistance training: the reproducibility and sensitivity of different velocity monitoring technologies. *Scientific Reports*. Email corresponding author: ivan.jukic@aut.ac.nz. Sport Performance Research Institute New Zealand (SPRINZ), Auckland University of Technology, Auckland, New Zealand.

**Supplementary File II**

Supplementary table S1. Missing observations for different devices across days and protocols

|  | Day 1 | | Day 2 | |
| --- | --- | --- | --- | --- |
| Protocol | 1RM | RTF | 1RM | RTF |
| Participants | 51 | 49 | 50 | 46 |
| Observations | 709 | 1351 | 694 | 1260 |
| Right GymAware (NA) | 0 | 0 | 0 | 1 |
| Right GymAware (NA) | 0 | 1 | 0 | 0 |
| Right PUSH2 (NA) | 0 | 52 | 4 | 20 |
| Left PUSH2 (NA) | 53 | 77 | 58 | 68 |
| Right Vmaxpro (NA) | 8 | 57 | 4 | 28 |
| Left Vmaxpro (NA) | 8 | 54 | 4 | 42 |

*Note:* 1RM, 1 Repetition Maximum; RTF, Reps to Failure; NA, Not Available
