# Supplementary material for: Implementing a velocity-based approach to resistance training: the reproducibility and sensitivity of different velocity monitoring technologies
Source: Sci Rep. 2023 May 2;13:7152. doi: 10.1038/s41598-023-34416-0 (PMC10154341; doi:10.1038/s41598-023-34416-0)
Supplement: Supplementary file 3 — Supplementary Information 3. [file 41598_2023_34416_MOESM3_ESM.docx]

Jukic et al. (2023). Implementing a velocity-based approach to resistance training: the reproducibility and sensitivity of different velocity monitoring technologies. *Scientific Reports*. Email corresponding author: ivan.jukic@aut.ac.nz. Sport Performance Research Institute New Zealand (SPRINZ), Auckland University of Technology, Auckland, New Zealand.

**Supplementary File III**

Supplementary table S2. Within-unit agreement and sensitivity estimators of GymAware, PUSH2 and Vmaxpro (EndorePro) devices.

| Measurement unit  Velocity metric | Estimator | Value | Lower limit | Upper limit |
| --- | --- | --- | --- | --- |
| GymAware  Mean Velocity | intercept (m/s) | 0.000 | -0.002 | 0.002 |
|  | slope | 1.001 | 0.996 | 1.005 |
|  | RSE (m/s) | 0.027 | 0.026 | 0.028 |
|  | r | 0.995 | 0.995 | 0.995 |
|  | PPER | 0.957 | 0.955 | 0.966 |
|  | SDC (m/s) | 0.054 | 0.052 | 0.056 |
|  | SDC%1RM | 4.418 | 4.193 | 4.506 |
| PUSH2  Mean Velocity | intercept (m/s) | -0.004 | -0.007 | 0.000 |
|  | slope | 1.012 | 1.006 | 1.018 |
|  | RSE (m/s) | 0.042 | 0.041 | 0.043 |
|  | r | 0.988 | 0.987 | 0.988 |
|  | PPER | 0.804 | 0.802 | 0.819 |
|  | SDC (m/s) | 0.082 | 0.080 | 0.085 |
|  | SDC%1RM | 6.687 | 6.398 | 6.779 |
| Vmaxpro  (EnodePro)  Mean Velocity | intercept (m/s) | 0.000 | -0.002 | 0.001 |
|  | slope | 1.000 | 0.996 | 1.003 |
|  | RSE (m/s) | 0.023 | 0.022 | 0.024 |
|  | r | 0.997 | 0.996 | 0.997 |
|  | PPER | 0.985 | 0.984 | 0.989 |
|  | SDC (m/s) | 0.044 | 0.043 | 0.046 |
|  | SDC%1RM | 3.607 | 3.431 | 3.686 |
| GymAware  Peak Velocity | intercept (m/s) | 0.011 | 0.006 | 0.017 |
|  | slope | 0.990 | 0.985 | 0.995 |
|  | RSE (m/s) | 0.051 | 0.050 | 0.052 |
|  | r | 0.989 | 0.988 | 0.990 |
|  | PPER | 0.777 | 0.773 | 0.797 |
|  | SDC (m/s) | 0.100 | 0.097 | 0.103 |
|  | SDC%1RM | 6.515 | 6.281 | 6.674 |
| PUSH2  Peak Velocity | intercept (m/s) | -0.002 | -0.009 | 0.005 |
|  | slope | 1.006 | 0.999 | 1.014 |
|  | RSE (m/s) | 0.075 | 0.073 | 0.077 |
|  | r | 0.979 | 0.977 | 0.980 |
|  | PPER | 0.675 | 0.672 | 0.695 |
|  | SDC (m/s) | 0.146 | 0.143 | 0.151 |
|  | SDC%1RM | 8.573 | 8.212 | 8.731 |
| Vmaxpro  (EnodePro)  Peak Velocity | intercept (m/s) | 0.011 | 0.005 | 0.017 |
|  | slope | 0.990 | 0.986 | 0.995 |
|  | RSE (m/s) | 0.056 | 0.055 | 0.058 |
|  | r | 0.988 | 0.987 | 0.988 |
|  | PPER | 0.740 | 0.734 | 0.762 |
|  | SDC (m/s) | 0.110 | 0.107 | 0.114 |
|  | SDC%1RM | 6.907 | 6.630 | 7.086 |

Note. RSE, Residual standard error; PPER, the proportion of practically equivalent residuals; SDC, the smallest detectable change; 1RM, One-repetition maximum; Upper- and Lower-limits represent bootstrapped 95% confidence intervals
