# Supplementary figures and images for: Implementing a velocity-based approach to resistance training: the reproducibility and sensitivity of different velocity monitoring technologies
Source: Sci Rep. 2023 May 2;13:7152. doi: 10.1038/s41598-023-34416-0 (PMC10154341; doi:10.1038/s41598-023-34416-0)

# *SESOI estimation for GymAware devices*

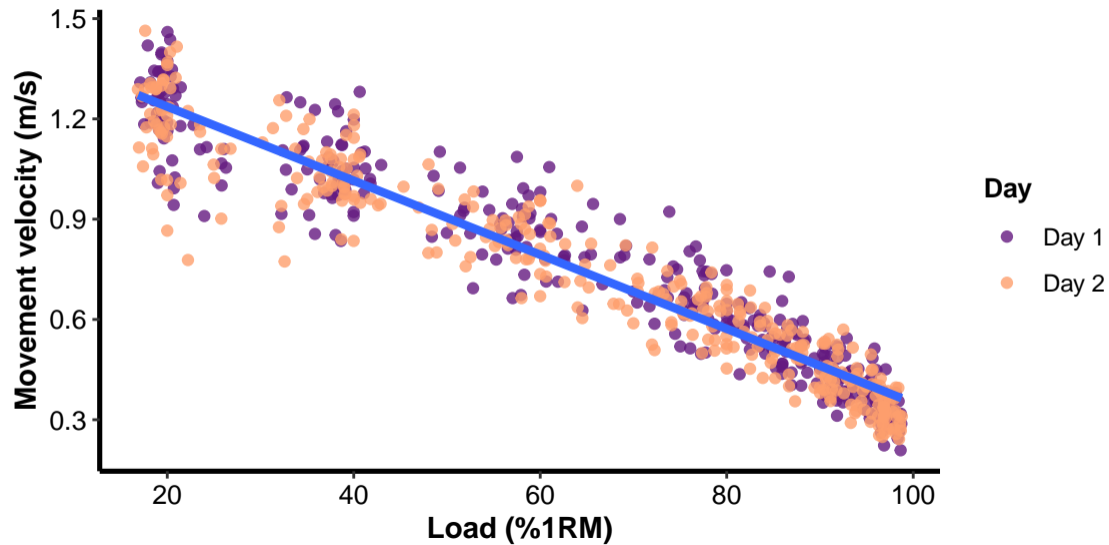

Supplement: Supplementary file 4 — Supplementary Information 4. [file 41598_2023_34416_MOESM4_ESM.pdf]
